# Navigating adolescence matters in Indonesia: insights and needs of students in Islamic schools

**DOI:** 10.11604/pamj.2025.50.100.46168

**Published:** 2025-04-11

**Authors:** Cennikon Pakpahan, Bella Amanda, Renny Sumino, Azlansyah Azlansyah, Christian Melka Simatupang, Lika Putri Handini, Tia Maya, Pranee Liamputtong, Andri Rezano

**Affiliations:** 1 Andrology Study Program, Faculty of Medicine, Universitas Airlangga, Surabaya, Indonesia,; 2 Department of Biomedical Sciences, Faculty of Medicine, Universitas Airlangga, Surabaya, Indonesia,; 3 Department of Forensic and Medicolegal, Faculty of Medicine, Universitas Airlangga, Airlangga, Indonesia,; 4 College of Health Sciences, Vin University, Gia Lam District, Hanoi, Vietnam,; 5 Center for Global Health, Perelman School of Medicine, University of Pennsylvania, Philadelphia, USA,; 6 Department of Biomedical Sciences, Faculty of Medicine, Universitas Padjadjaran, Kabupaten Sumedang, Indonesia

**Keywords:** Adolescence, education, puberty, reproductive health, qualitative study

## Abstract

**Introduction:**

adolescence is a critical period marked by significant physical, emotional, and social changes. In Indonesia, where cultural and religious norms play a central role in shaping societal attitudes, adolescents face unique challenges as they navigate through this developmental stage. Islamic boarding schools, or pesantren, provide a distinctive setting where adolescents receive secular and religious education. Understanding the experiences and needs of adolescents in these settings is essential for developing targeted interventions to support healthy development.

**Methods:**

this study employed a qualitative approach, utilizing focus group discussions to explore students' perspectives in Islamic boarding schools. Purposive sampling was used to recruit 32 participants, divided into male and female groups. Data were collected through recorded interviews, transcribed, and analyzed thematically.

**Results:**

three main themes emerged from the data: the importance of reproductive health knowledge, navigating romantic feelings and privacy, and the impact of bullying. Cultural and religious values were identified as significant influencers of adolescent behavior, highlighting the importance of integrating these values into educational interventions. They also discussed challenges related to romantic feelings, privacy boundaries, and bullying experiences within the school environment.

**Conclusion:**

this study provides valuable insights into the experiences and needs of adolescents in Islamic boarding schools in Indonesia. The findings emphasize the importance of comprehensive sex and reproductive education, cultural sensitivity, and targeted interventions to support adolescent well-being in these settings. Future research should explore the perspectives of parents and educators to develop more holistic interventions.

## Introduction

Adolescence is a pivotal period of human development characterized by a series of significant physical, emotional, and psychological changes. As individuals transition from childhood to adulthood, they undergo puberty, which brings about rapid growth and the development of secondary sexual characteristics. This phase of life is marked not only by biological maturation but also by the formation of personal and social identities, including the exploration of sexual and reproductive health [1]. Adolescents often face heightened vulnerability during this transformative time due to their evolving cognitive abilities and emotional sensitivities [2]. A key challenge during adolescence is the quest to understand and manage their emerging sexual and reproductive health, exposing young people to various risks and challenges such as sexually transmitted infections (STIs), unintended pregnancies, and societal pressure regarding sexual behavior [3]. Many adolescents lack access to accurate information and comprehensive education about sexual and reproductive health, further exacerbating their vulnerability. In today´s digital era, adolescents frequently turn to their peers and digital media for information and guidance, where peer pressure and the desire to fit in can compel them to participate in risky activities, such as cybersex [4].

Understanding these vulnerabilities, including sexual and reproductive health, potential harassment, and the dynamics of being either a victim or bully, is essential. Credible and reliable sex education is critical. In Indonesia, cultural and religious norms often render discussion about sexuality and reproductive health taboo in families [5]. Conflicting messages about sexuality are common in Indonesia, a predominantly Muslim country with deeply rooted cultural and religious values. Conservative norms emphasize modesty and abstinence, leading to limited open discussion about sexual health, while the pervasive nature of the internet exposes adolescents to a plethora of unfiltered and often inaccurate information about sex and relationships [6]. Despite UNESCO´s inclusion of gender-responsiveness, life skills-based HIV and sexuality education in the secondary education curriculum [7], sexual and reproductive health services are predominantly accessible only to married couples [8]. This discrepancy between the curriculum and real-world accessibility fails to meet the evolving needs of Indonesian adolescents.

Technological advances have made it easy for adolescents to access information, creating generational differences in how they respond to changes [9]. Generation Z, in particular, frequently uses digital technology for information, valuing its flexibility [10]. However, this convenience can lead to risky behaviors if not properly guided [11]. Moreover, the phenomenon of cybersex among teenagers contributes to issues such as teenage promiscuity, pre-marital sexuality, and increased sexual abuse. In 2014, the Indonesian Child Protection Commission (KPAI) noted that 90% of perpetrators of sexual violence against children in Flores were influenced by pornographic content, highlighting the prevalence of pornography among adolescents [11]. In addition, the National Commission for the Protection of Women and Children has recorded 15,210 cases of violence against women and children in 2023, which includes sexual violence against children [12]. Clearly, there is a pressing need for credible and appropriate information transfer to help adolescents make informed decisions.

Islamic teachings emphasize social responsibility and accountability in sexual matters, contrasting with the Western focus on individual rights [13]. Islamic sex education focuses on ethics, modesty, worship, and avoiding negative influences. It includes teaching about puberty and maintaining open communication with parents to provide age-appropriate guidance [14]. Sexuality is considered a private matter associated with shame and pornography, which discourages open discussion [15]. Understanding puberty and its implications, as studied by Sutanto *et al*. [16], Patroni *et al*. [17], and [18], is crucial for reducing cases of sexual harassment, unwanted pregnancies, and bullying. Thus, comprehensive sex education in Indonesia should blend religious doctrine with scientific evidence to create culturally sensitive yet effective programs [19]. Therefore, the perspective of adolescents in Islamic boarding schools offers valuable insights.

Cases of child sexual abuse are common among teenagers who also live in dormitories like pesantren. Sometimes, adolescents' understanding of significant changes, especially sexual and reproductive organs that are not yet complete, causes them to be vulnerable to various cases of abuse. Given the generational differences and rapid societal changes, it is essential to determine the appropriate approach and depth of information for adolescents. This study aims to provide a nuanced perspective by exploring the qualitative views of Generation Z on their evolving lives, combined with their cultural and religious backgrounds as Indonesian teenagers. This research aligns with the Sustainable Development Goals (SDGs), especially SDG 3 (Good Health and Well-being), SDG 4 (Quality Education), and SDG 5 (Gender Equality) and underscores the importance of effective sexual and reproductive education [20,21].

## Methods

**Study design:** this study employed a qualitative approach [22], specifically a descriptive qualitative design, utilizing focus group discussion (FGD) to gather the perspectives and experiences of respondents. This is essential, as we know little about the issues we examined in this study [20].

**Settings:** the respondents were students from an Islamic boarding junior high school in Jombang, East Java, Indonesia. The reason for choosing Jombang as the location of this research is that Jombang is known as one of the most religious areas in East Java and is often known as a pesantren area. So, the selection of this pesantren school is expected to represent Muslims.

**Samples/participants:** they were recruited through purposive sampling in accordance with the provisions of qualitative studies. They divided into two groups based on gender due to the school´s policy of segregating boys and girls. All respondents were required to be proficient in Bahasa Indonesia. The FGD was facilitated by experienced interviewers (authors 1, 2, 3, and 4) who posed questions from a predetermined list (Table 1). The discussions continued until data saturation was achieved [20]. The sample included in this study was boarding school students in grades second and third year who could communicate well, were willing to join the discussion, and were pubescent. To avoid triggering trauma, we did not include students who were victims of harassment or had experienced it.

**Table 1:** list of questions and probe

Guide questions	Probe
What do you think puberty is?	How would you describe puberty? Did you go through puberty? When did you experience it?
How do you feel about the changes in your body?	How are your attitudes, especially with the changes in sensitive areas? How do you respond to the changes in your attitude and mindset currently? Has anyone started to become sexually attracted? What did you feel when you became sexually attracted?
What is your opinion on early marriage?	What do you think of when asked when to get married? When should you get married?
How have you been getting information about sexual and reproductive health?	Did you get it from school? Can you tell us? What do you think about learning sexual and reproductive health at school? How should you know about sexual and reproductive health information at this age? What do you think is a valid and reliable source of information about sexual and reproductive health?
What does privacy mean for you?	What are some areas of privacy for you right now? How do you maintain your privacy?
What do you think about cases of bullying, both verbally and physically?	What is bullying? What do you do if you see your friends being bullied, both verbally and physically?

**Data collection:** the focus group discussion (FDG) method was used for data collection. Each FGD session lasted between 45-60 minutes and was recorded using a mobile phone. The discussions were conducted in Bahasa Indonesia. Before participation, each respondent signed an informed consent form with the guidance of a teacher. The FGD lasted approximately 45-60 minutes. The researcher led the discussions, with assistance from two other facilitators to ensure a conducive environment. Before the discussions, the discussion leader briefed the participants about the purpose of the study and the specific questions that would be asked. The discussions were recorded, transcribed, and confirmed with the school before further analysis. Data collection occurred on July 30, 2023.

**Data analysis:** data were thematically analyzed. This process began with familiarizing ourselves with the data, generalizing initial codes, searching and reviewing themes, defining themes, and finally writing the theme. The study´s reporting adhered to the Consolidated Criteria for Reporting Qualitative Research (COREQ) Checklist.

**Trustworthiness:** several measures were implemented to ensure the validity of the research data [23]. These included triangulation and member checking. The research questions and procedures were developed during regular researcher meetings. Initial visits and surveys were conducted to establish rapport with the school and participants. Informed consent was obtained with the assistance of teachers, who explained the purpose of participation. Interviews were conducted privately to encourage openness, recorded, and subsequently transcribed. The transcription involved documenting all respondents' statements. The collected data were first discussed among the researchers before being submitted to independent coders for peer debriefing and further analysis. The final analysis was shared with the school for feedback before being compiled into the research report.

**Ethical considerations:** the research is part of the Faculty of Medicine community service initiative and was conducted at an Islamic boarding junior high school in Jombang, East Java, Indonesia. Ethical approval was granted by the Health Research Ethics Committee, Faculty of Medicine Universitas Airlangga, with reference number 277/UN3.15/PM/2023. These ethics have been adapted to the provisions of national and international ethical standards for research involving minors such as adolescents. Consent to become a respondent involves the counselling teacher in communicating with the student, but in the FGD process, the teacher is not involved in encouraging the respondent's openness.

## Results

**Figure 1:**
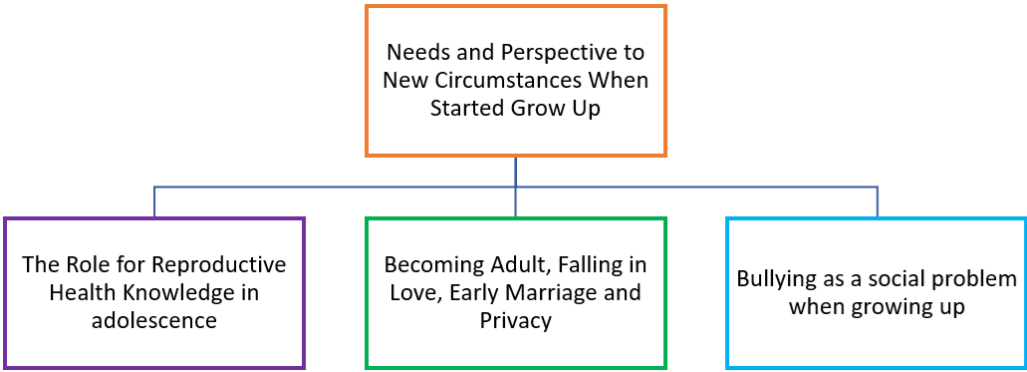
theme analysis

A total of 32 students participated in the study, divided into two FGDs: one for males and one for females, following the rules of the Muslim boarding school. The female group consisted of 18 participants (56.25%), and the male group consisted of 14 participants (43.75%). The mean age of the participants was 14 years, with the oldest being 15 and the youngest being 12. All participants provided informed consent before participating in the discussions and interviews, with guidance from the school counselling teacher. From the analysis of the FGD, three major themes emerged, each with several sub-themes and categories. These themes reflect the participants´ perspectives on various aspects of adolescence, including reproductive health, personal development, and social challenges. The themes are illustrated in Figure 1, and detailed descriptions are provided in Table 2, Table 3, and Table 4.

**Table 2:** theme 1: role for reproductive health knowledge in adolescence

Categories	Codes	Quotes
Knowledge about puberty and adolescent reproductive health	The benefit of good understanding	For me, by knowing my reproductive organs and those of the opposite gender, I can take better care of genital hygiene, avoid potential diseases, and also understand how to use them at the right time (K, boy-14 years old).
	Knowledge and many kinds of information on teenagers' reproductive health	Learning about reproductive organs and how they function. Also learning about the meeting of sperm and egg (F, boy-14 years old). According to me, I already feel sufficiently helped by that explanation (in 6th grade), from initially not knowing to becoming informed (Z, girl-12 years old).
Sources of reproductive health information for adolescents	Family as the source of information	I learned information about reproductive organs from a cousin who has already gone through puberty (N, girl-12 years old).
	Lessons from school as the source of information	In school, there is education about puberty starting in the 6th grade (S, girl-13 years old).
	Social media as a source of information	The first time got information from social media, it appeared on the social media timeline (S, girl-14 years old).
A trusted source of reproductive health information for adolescents	General practitioners as a source of reliable information	In my opinion, the trustworthy source is directly from the doctor (N, girl-12 years old).
	Teachers as a source of reliable information	Beside general practitioner, teachers are also a good source of information (N, girl-12 years old).
	Family as a source of reliable information	In my opinion, it could be from a doctor or parents, but in my opinion, it's more about parents (A, girl-12 years old).
	Social media as a source of reliable information	...also, social media because I often scroll through it (R, girl-15 years old).
Expectations and barriers related to the source of information on adolescent reproductive health	Role of Information about reproductive health	How to take care of reproductive organs and how to identify abnormalities such as sores in our genital organs. It's also important to know about the reproductive organs of the opposite gender, as understanding their organs allows us to be more caring towards them (K, boy-14 years old).
	Lack of information about reproductive health	..however, in my opinion, currently at school, information about reproductive health, especially regarding the reproductive organs of the opposite gender, is still lacking. This includes understanding how one can become pregnant (K, boy-14 years old). In my opinion, it's not sufficient because there is a need for insight in dealing with that puberty period (S, girl-14 years old).
	Lack of knowledge about reproductive health	..Because experiencing the first menstruation, I don't know how to handle it (S, girl-14 years old).
	The use of language that is not too scientific or vulgar	It should not use language that is too vulgar because there are some words that are difficult to understand (A, girl-14 years old).

**Table 3:** theme 2: becoming adult, falling in love, early marriage, and privacy

Categories	Codes	Quotes
Desire to approach the opposite sex	Environment rules regarding dating	... in the dormitory or at school, indeed, dating is not allowed (U, boy-13 years old).
	Desire to approach the opposite sex	I support and endorse getting closer, in the dormitory or at school, indeed dating is not allowed (U, boy-13 years old).
	Adolescent perspectives on marriage and early marriage	If it's just for reproductive needs, maybe getting married is allowed, but because marriage involves other needs, such as economic and life necessities, it may not be sufficient or stable. Maybe around the age of 25 is the most appropriate time (K, boy-14 years old). We can get married after being financially stable and having a job (N, boy-14 years old).
Adolescent perspective on privacy	Definition of privacy	In my opinion, privacy is personal data that should only be known to ourselves. If others want to know, there must be our consent (Z, girl-12 years old). Privacy is also something that everyone needs. In boarding school, everyone has different personalities. Some like to expose other people's privacy or flaws, so it's like their privacy is less hidden, less protected (B, girl-14 years old).
	Kinds of privacy	Like, maybe a diary, right? It expresses feelings that we may not be confident about (R, girl-15 years old). People are not allowed to touch our genital area; only those who are mahram (permissible to marry) are allowed. Perhaps parents can touch our private parts. Only a mahram is allowed (K, girl-14 years old).
	Preventing privacy for adolescents	Everyone has privacy within themselves; maybe a person is shy to expose it. Personal issues shouldn't be broadcast (N, girl-14 years old). If you can't handle it, you should talk about it, but to someone trustworthy, like family or close friends (A, girl-12 years old). For personal issues, like if there's a problem, it's better to talk to Allah because Allah is the place to lean on (S, girl-14 years old).
	Many kinds of problems related to privacy	Sometimes they (others) tend to inform that this is privacy, but they belittle it, like, 'Oh, it's just this (A, girl-14 years old).

**Table 4:** theme 3: bullying as a social problem when growing up

Categories	Codes	Quotes
Bullying incidence from an adolescent perspective	An example of bullying behaviour	Saying offensive words" (N, boy-14 years old). Touching the genitals without permission (H, boy-14 years old).
	Perspective that there’s a reason for bullying behaviour	… but sometimes there's something that makes someone resort to bullying, so sometimes the victim also needs to understand why they have been bullied all this time (N, girl-12 years old).
	Perspective related to bullying behaviour	In my opinion, people who bully are mean, they don't have any sympathy for their victims, they don't reflect on themselves, they just bully. If the issue is small, why can't it be discussed calmly? If it's a joke, it shouldn't be taken too far (Z, girl-12 years old).
	Perspective related to the consequences of bullying.	Regarding bullying, in my opinion, it is not allowed because it can make the mental state of the bullied child go down, and in the end, they may become depressed (P, girl-12 years old).
Adolescent behaviour toward bullying	Desire for follow-up on reports of bullying cases	... If there's no clear reason, just for fun, it's better to report it to the teacher because it can harm the mental well-being of the person being bullied (P, girl-12 years old).
	Behaviour of protecting and intervening in bullying actions.	I see it and feel sorry. Those who bully don't think about what might happen in the future, so I try to calm down the one being bullied first (B, girl-14 years old).
	Attitude towards bullying behaviour	Actually, we can avoid bullying in friendships. If there's someone who doesn't like us, we can talk to them nicely (L, girl-14 years old).

**Theme 1: role of reproductive health knowledge in adolescence:** the first theme that emerged from the participant´s discussion centred around the role of reproductive health knowledge during adolescence. This theme comprised four distinct categories, as outlined in Table 2 participants emphasized the crucial role of knowledge in navigating the changes and developments experienced during adolescence. They expressed the need for comprehensive information regarding reproductive health to be readily available, highlighting the importance of this knowledge for their well-being. Participants identified various sources of information about reproductive health, including family, schools, and social media. However, they noted that these sources often fell short of providing the necessary information they desired. Specifically, participants expressed a need for more detailed information about reproductive organs, pregnancy prevention, and interpersonal relationships. They emphasized the importance of receiving accurate and accessible information, particularly when encountering significant life changes such as menstruation. Interestingly, despite their familiarity with digital technology, participants acknowledge the limitations of relying solely on online sources for reproductive health information. Instead, they emphasized the importance of seeking guidance from trusted professionals, such as doctors, who could provide accurate information clearly and understandably. Overall, the findings highlight the participants´ recognition of the importance of reproductive health knowledge during adolescence and underscore the need for accessible and reliable information to support their well-being during this critical stage of development.

**Theme 2: becoming an adult, falling in love, early marriage, and privacy:** puberty brings about significant changes in teenagers, including hormonal fluctuations that impact mood and emotions. Participants in our study discussed various concerns related to puberty, as summarized in Table 3. Notably, they highlighted the emergence of feelings of attraction towards the opposite sex despite residing in a boarding school environment guided by Islamic principles. Strict rules regarding gender interactions in dormitories require teenagers to learn to navigate these boundaries. Participants acknowledge the importance of controlling their emotions and adhering to the rules surrounding opposite-sex relationships. While the concept of attraction may initially be unfamiliar to them, they expressed confidence in their ability to manage these feelings with guidance. Furthermore, participants emphasized the distinction between attraction and marriage, asserting that marriage should occur only when individuals are emotionally and financially mature. Many participants suggested that the ideal age for marriage is over 25, emphasizing the importance of personal readiness and independence. Privacy emerged as another significant concern for teenagers, particularly regarding their genital organs. Participants stressed the importance of respecting personal boundaries and privacy, advocating for limitations on who can access intimate body parts. While they recognized the necessity of privacy, participants also acknowledged the challenges of adolescence and the importance of having a trusted individual to confide in. This perspective on privacy was strongly influenced by cultural and religious values, underscoring the role of religion in shaping their attitudes towards personal boundaries.

**Theme 3: bullying as a social problem during adolescence:** participants also discussed their experiences with bullying, highlighting it as a pervasive issue within their environment, as outlined in Table 4. They condemned bullying as a reprehensible act that manifests in various forms, including verbal abuse and physical violence. Despite recognizing the harmful effects of bullying on victims´ mental health, participants emphasized the need to understand the motivations behind the perpetrator´s actions. While condemning bullying, participants advocated for a nuanced approach to addressing the issue, emphasizing the importance of dialogue and conflict resolution. They stressed the need for both perpetrators and victims to receive support and intervention from the school community. Participants recognized the significant impact of bullying on victims´ well-being and encouraged reporting incidents to school authorities as a proactive step towards addressing the problem. Overall, participants´ discussion on puberty and bullying shed light on the complex social and emotional challenges faced by adolescents, underscoring the importance of supportive environments and effective communication in promoting adolescent well-being.

## Discussion

Our study provides insights into several key issues faced by adolescents as they navigate puberty. These issues include the need for comprehensive sex and reproductive education, understanding and setting boundaries for privacy, and dealing with bullying. Our findings highlight the critical role of accurate and accessible information in helping adolescents adapt to these changes, emphasizing the importance of professional guidance. The need for detailed and reliable sex and reproductive education emerged as a prominent theme. Despite the pervasive use of digital devices among Generation Z, our participants expressed a preference for information from professionals over online sources. This preference underscores a significant gap in the accessibility and quality of online educational resources on reproductive health. Adolescents recognize the complexity and sensitivity of this information, which necessitates a professional´s expertise to ensure accuracy and comprehensibility. This aligns with the WHO´s standards for age-appropriate sex education, which emphasize the role of tailored and professional guidance [20].

The reluctance to rely solely on the internet is also influenced by the prevalence of distorted and unrealistic portrayals of sexual content online, often degrading women. Professional guidance provides a more balanced and respectful understanding of sexual and reproductive health. However, in Indonesia, sex education remains a taboo topic, often avoided in family and school settings due to cultural and religious beliefs [5,24]. Some parents even argue that teaching sex education is the same as teaching them to have sex [24]. Our study did not interview the parents of the participating students. This absence may have overlooked the influence of parental attitudes and beliefs on adolescents´ perceptions and behaviors regarding sexual and reproductive health. Future studies should consider including parental interviews to obtain a more comprehensive understanding of adolescent development within the family context. Our study highlights adolescents´ growing awareness of privacy, particularly regarding their bodies and personal information. Participants emphasized the need for clear boundaries, recognizing the importance of privacy in their developmental stage. This understanding is crucial in preventing sexual abuse and harassment, as ignorance of the boundaries can lead to victimization [25]. Interestingly, the views on who is allowed to touch their genital organs varied, reflecting a blend of personal beliefs and cultural teachings. The minimal use of cellular phones in their dormitory setting positively impacted their understanding of online privacy, reducing the risk of privacy breaches. This finding supports the framework proposed by Wisniewski *et al*. [26], which advocates monitoring and restricting online activities to safeguard adolescents´ privacy.

Adolescents in our study expressed a nuanced understanding of romantic relationships and the implications of early marriage. While they acknowledged the natural emergence of romantic feelings during puberty, they also recognized the dormitory´s strict rules against dating. Puberty is a phase in human life characterized by a significant surge in sex hormones, and it is very natural to desire a romantic relationship at their age, as stated by a study by Ali *et al*. [27] and Romeo *et al*. [28]. A study by Ali *et al*. [27] supports our findings by highlighting how young Muslims navigate the intersection of modern technology and traditional values when it comes to dating. This adaptation reflects their ability to navigate personal desires within the constraints of their environment, guided by strong religious values. Despite the pervasive influence of digital communication and social media platforms, religious teaching and cultural norms still play a crucial role in guiding the behavior of adolescents, especially within Muslim communities.

The phenomenon of early marriage in Indonesia, where one in nine girls is married before the age of 18, underscores the urgency of comprehensive sex and reproductive education [29]. Participants argued that emotional and financial maturity are essential for marriage, opposing the idea of early marriage, which is often driven by economic and educational factors [30]. Sometimes, early marriage does not come from the teenagers' wishes but from the parents. Under these conditions, parents are also targeted for sex and reproductive education [31]. Unfortunately, we did not interview parents to confirm this. This perspective highlights the critical role of education in empowering adolescents to make informed decisions about their futures. Bullying emerged as a significant concern among adolescents, with participants describing various forms, including physical, verbal, and social bullying. They recognized the severe long-term impacts of bullying on mental health and advocated for appropriate interventions to address this issue. This understanding aligns with previous research that highlights the detrimental effects of bullying on victims´ psychological well-being [32,33].

## Conclusion

Our study elucidates that adolescence is a transformative phase filled with challenges and new experiences. Generation Z exhibits a pragmatic approach to essential issues, balancing modern influences with traditional values. The generation's cautious and informed perspective on sex education, privacy, romantic relationships, and bullying reflects a maturity that bodes well for their future. However, this study has limitations, including the need for a deeper exploration of the role of religious values and a broader examination of adolescents' experiences in boarding school settings. However, we also admit that it is very difficult to generalize the findings in this study directly to represent the perspective of Generation Z, who live in pesantren, so future research is needed in a broad scope, including different ethnic groups and social stratification. We also suggest further studies should address these aspects to provide a more comprehensive understanding of adolescents' readiness for puberty and the support system necessary for their development.

### What is known about this topic

- Culture and religious values influence the perspective and understanding of adolescents in dealing with issues, especially sexual and reproductive health issues;
- Indonesia with its diversity of ethnicity, religion and race is often considered closed in dealing with sexual and reproductive health issues which are important in adolescence;
- Lack of education and information about adolescents is a major target in health education, especially sex education.

### What this study adds

- Our study adds a new perceptive of students living in a dormitory with strong Islamic religious values so that it can be a reference in determining health education strategies, especially sex in Islamic adolescents;
- This study involved participants from generation Z who are known to be active on social media and the internet in digging up information, but on the one hand, it turns out the easy availability of information does not make them easy to understand, providing health information, especially sexual and reproductive health information.

## References

[1] Committee on the Neurobiological and Socio-behavioral Science of Adolescent Development and Its Applications (2019). Board on Children, Youth, and Families, Division of Behavioral and Social Sciences and Education, Health and Medicine Division, National Academies of Sciences, Engineering, and Medicine. The Promise of Adolescence: Realizing Opportunity for All Youth.

[2] UNICEF (2011). Adolescence: An Age of Opportunity.

[3] Morris JL, Rushwan H (2015). Adolescent sexual and reproductive health: The global challenges. Int J Gynaecol Obstet.

[4] Widman L, Choukas-Bradley S, Helms SW, Prinstein MJ (2016). Adolescent Susceptibility to Peer Influence in Sexual Situations. J Adolesc Health.

[5] Situmorang A (2003). Adolescent reproductive health in Indonesia. Jakarta: STARH Program.

[6] Weintraub AN (2011). Islam and popular culture in Indonesia and Malaysia.

[7] Joint United Nations Programme on HIV/AIDS (2021). The journey towards comprehensive sexuality education: global status report.

[8] Chandra-Mouli V, Plesons M, Hadi S, Baig Q, Lang I (2018). Building Support for Adolescent Sexuality and Reproductive Health Education and Responding to Resistance in Conservative Contexts: Cases From Pakistan. Glob Health Sci Pract.

[9] Berge ZL, Berge MB (2019). The economic ABCs of educating and training generations X, Y, and Z. Performance Improvement.

[10] Januariyansah S, Rohmantoro D (2018). The role of digital classroom facilities to accommodate learning process of the Z and Alpha Generations. In The 2^nd^ International Conference On Child-Friendly Education (ICCE).

[11] Makaria EC, Rachmayanie R, Adawiyah R (2021). Teenagers' promiscuity of alpha generation. In2nd International Conference on Social Sciences Education (ICSSE 2020).

[12] Paudpedia 15,120 Cases of Violence Against Women and Children Occurred in 2023 Report Violence on WhatsApp Service SAPA 129 (2024). PAUDPEDIA - 15.120 Kasus Kekerasan Perempuan dan Anak Terjadi Tahun 2023, Laporkan Aksi Kekerasan di Layanan WhatsApp SAPA 129.

[13] Ihwani SS, Muhtar A, Musa N, Yaakub A, Mohamad AM, Hehsan A (2017). An overview of sex education: Comparison between Islam and western perspectives. Al-Qanatir: International Journal of Islamic Studies.

[14] Desiningrum DR (2018). Sexual Education For Children With Islamic Psychological Approach. In Proceeding Annual International Conference on Islam and Civilization.

[15] Putra PH, Erniyati Y (2022). Sex Education in Islamic Education Perspective. AJIS.

[16] Sutanto NH, Utami E, Rismayani R (2021). Systematic literature review untuk identifikasi metode evaluasi website layanan pendidikan di indonesia. Jurnal Ilmiah IT CIDA.

[17] Patroni R (2019). The effect of sex education on youth knowledge about sexual behavior in storage in sma negeri 2 kaur. In1st International Conference on Inter-Professional Health Collaboration (ICIHC 2018).

[18] The Relationship Between Sex Education And Sexual Behavior In Female Adolescents At State Senior High School 4 Binjai In 2017 (2018). Hubungan Pendidikan Seks Dengan Perilaku Seksual Pada Remaja Putri Di Sma Negeri 4 Binjai Tahun 2017. *Prodi Kesehatan Masyarakat Fakultas Kesehatan Masyarakat UIN Sumatera Utara*.

[19] Horanieh N, Macdowall W, Wellings K (2020). Abstinence versus harm reduction approaches to sexual health education: views of key stakeholders in Saudi Arabia. Sex Education.

[20] WHO BZga (2010). Standards for Sexuality Education in Europe.

[21] United Nations THE 17 GOALS | Sustainable Development.

[22] Liamputtong P, Ezzy D (2005). Qualitative research methods.

[23] Lindgren B-M, Lundman B, Graneheim UH (2020). Abstraction and interpretation during the qualitative content analysis process. Int J Nurs Stud.

[24] Zakiyah R, Prabandari YS, Triratnawati A (2018). Taboo, a cultural barrier to early sexuality education for children in Dumai City. Tabu, hambatan budaya pendidikan seksualitas dini pada anak. BKM Journal of Community Medicine and Public Health.

[25] Hidayati WR, Nurhafizah N (2022). Introduction of sex education to early childhood: to reduce cases of child sexual abuse. Indonesian Journal of Early Childhood Education Studies.

[26] Wisniewski PJ, Vitak J, Hartikainen H (2022). Privacy in Adolescence. Modern Socio-Technical Perspectives on Privacy.

[27] Ali N, Phillips R, Chambers C, Narkowicz K, Hopkins P, Pande R (2020). Halal dating: Changing relationship attitudes and experiences among young British Muslims. Sexualities.

[28] Romeo RD, Richardson HN, Sisk CL (2002). Puberty and the maturation of the male brain and sexual behavior: recasting a behavioral potential. Neuroscience & Biobehavioral Reviews.

[29] UNICEF (2022). 1 of 9 Indonesian Women Are Married Before They Turn 18.

[30] Angkasa AB (2021). Early Marriage Problems in Indonesia. Semarang State University Undergraduate Law and Society Review.

[31] Desiyanti IW (2015). Factors Related to Early Marriage in Fertile Age Couples in Mapanget District, Manado City. Faktor-Faktor yang Berhubungan Terhadap Pernikahan Dini Pada Pasangan Usia Subur di Kecamatan Mapanget Kota Manado. E-Journal UNSRAT.

[32] Tommola P, Unkila-Kallio L, Paavonen J (2010). Surgical treatment of vulvar vestibulitis: a review. Acta Obstet Gynecol Scand.

[33] Kaltiala-Heino R, Rimpelä M, Rantanen P, Rimpelä A (2000). Bullying at school-an indicator of adolescents at risk for mental disorders. Journal of Adolescence.

